# Surgery of the Pericardium

**Published:** 1891-07-25

**Authors:** 


### SURGERY.

### SURGERY OF THE PERICARDIUM.

In our issue for July 4th we drew attention to recent advances in the surgical treatment of the pericardium in reference, more especially, to Dr. Bronner's case of incision of the pericardium and drainage for purulent pericarditis. Dr. Samuel West,* commenting on this case, furnishes some interesting remarks on the treatment of purulent pericarditis. The dangers of surgical interference with the pericardium are greatly exaggerated. The only risk, wounding the heart, may with care be avoided. Exploratory punctures and paracentesis are much more risky than incision. He has tapped the pericardium several times, and without a single misadventure. On one occasion he was alarmed by getting nothing but a jet of blood. He at once removed the needle, but the patient was greatly relieved and rapidly improved. The chief risk is when the diagnosis is incorrect, and the difficulties are in some cases considerable in distinguishing between a greatly distended heart and pericardial effusion. Even then it is a laceration, not a clean puncture through the walls of the heart that is dangerous, for the latter has frequently occurred as the result of accident, and has even been deliberately performed with the object of tapping a greatly distended cavity. The dangers, again, of the rapid removal of fluid are, Dr. West believes, for the most part imaginary. In his published case the incision was followed by a gush of pus, which spurted out eight or ten inches from the chest; in a few seconds fully two quarts were evacuated, and that without any bad symptoms or even faintness; on the contrary, with immediate relief. Washing out the pericardium also inspires much fear in the minds of some writers, and with little real ground. In his case the pericardium was many times washed out, and always without harm. Dr. West further records the fact that his patient, whose pericardium was opened, washed out, and drained, has developed into a full-grown man, above the average weight and height. Although the pericardium must be universally adherent, there are no physical signs to indicate this. He has for years been in active employment, and is capable of exertion in ordinary muscular work. Such cases as these must come to many practitioners as something entirely new in their medical experience, but simply go to prove that prejudice has too long stood in the way of their rendering useful aid to their patients *in extremis*.

